# Clinicians’ perspective of picture archiving and communication systems at Charlotte Maxeke Johannesburg Academic Hospital

**DOI:** 10.4102/sajr.v27i1.2578

**Published:** 2023-05-10

**Authors:** Polite Tshalibe, Jacinta Adrigwe, Susan Lucas

**Affiliations:** 1 Department of Radiology, Faculty of Health Sciences, Rahima Moosa Mother and Child Hospital, University of the Witwatersrand, Johannesburg, South Africa; 2 Department of Radiology, Faculty of Health Sciences, Highveld Hospital, University of the Witwatersrand, Johannesburg, South Africa; 3 Department of Radiology, Faculty of Health Sciences, Chris Hani Baragwanath Academic Hospital, University of the Witwatersrand, Johannesburg, South Africa

**Keywords:** picture archiving and communication systems (PACS), physicians, cross sectional studies, surveys and questionnaires, South Africa, referral and consultation, clinician satisfaction

## Abstract

**Background:**

Picture archiving and communication systems (PACS) are now an established means of capturing, storing, distribution and viewing of all radiology images. The study was conducted in a quaternary hospital, Charlotte Maxeke Johannesburg Academic Hospital (CMJAH), part of the University of the Witwatersrand teaching circuit, in South Africa.

**Objectives:**

To measure the clinicians’ perceived benefits and challenges of PACS. To document perceived views on how the current PACS can be improved.

**Method:**

This was a cross-sectional observational study over a period of 5 months from September 2021 to January 2022 carried out at CMJAH. Questionnaires were distributed to referring clinicians with PACS experience. Descriptive statistics was conducted. Categorical variables were presented as frequency and percentages. The continuous variables were presented as means ± standard deviation.

**Results:**

A survey with a response rate of 54% found the benefits most reported by clinicians were improved patient care, less time needed to review an exam, improved image comparison and consultation efficiency. With respect to perceived challenges, the unavailability of images at the bedside, problems with access and the lack of advanced image manipulating software were noted. The most frequent recommendations on improvements focused on the aforementioned challenges.

**Conclusion:**

Hospital-wide PACS was viewed beneficial by most clinicians. Nonetheless, there are a few aspects that deserve attention to improve the functionality and access of the system.

**Contribution:**

The findings will assist in future hospital or provincial-wide PACS deployment projects.

## Introduction

Picture archiving and communication systems (PACS) are now an established, recognised and appropriate means of digital image acquisition, archiving, distribution and viewing.^1^ The technology is unique in that it delivers the radiology diagnostic images and reports to the clinicians at the point of care.^2^

Picture archiving and communication systems present an opportunity to eliminate film-based imaging.^2,3^ In the past, the means for capturing, storing and viewing medical images was the hard copy film.

The last few years have seen a tremendous increase in the adoption of PACS in most radiology departments in South Africa.^4^ The implementation of PACS began primarily in the private sector, with the public sector implementation of PACS significantly lagging behind because of lack of funding.^4^ Currently, most public sector hospitals in South Africa have a mini PACS limited to the radiology department. A hospital-wide PACS was first installed in 2016 at the Charlotte Maxeke Johannesburg Academic Hospital (CMJAH). The onsite-PACS network was set up by Phillips architects, who configured the system connecting all the radiology imaging hardware, radiology and clinician workstations, using a software called iSite.

It has been well documented in the literature that hospital staff do not easily accept new technology unless they understand and embrace it fully.^5^ The focus of the present study was to evaluate the benefits and challenges clinicians perceived from the PACS at CMJAH, 6 years post-implementation of the system. In addition, their views on how the system could be improved were documented.

## Materials and methods

### Study design

A cross-sectional, observational, descriptive study design based on a questionnaire survey was followed. A pre-tested questionnaire used by Jorweker et al.^1^ was adopted and modified to suit our local environment. A four-point Likert scale and a categorical approach were used to elicit responses for the majority of statements. Responses to statements ranged from 1 to 5: 1 (strongly disagree) to 4 (strongly agree) and 5 (Neutral). Some opportunities for open-ended questions were included.

### Study setting

The study population included referring doctors from different specialities who routinely referred patients to the radiology department for imaging. Interns, medical officers, registrars and consultants were among them. Further included were the registrars who had rotated through CMJAH from other university circuit hospitals. Radiologists and radiology registrars were excluded. The hospital is a quaternary government hospital situated in Johannesburg, in the Gauteng Province of South Africa.

### Data collection

Data collection was performed over a 5-month period from 01 September 2021 to 31 January 2022. A sample size of 375 was calculated with the open source epidemiologic statistics calculator for public health^6^ using a power of 80% at 0.05 alpha with a 95% confidence interval. Convenience sampling of the referring clinicians with PACS experience at the academic hospital resulted in the distribution of 682 questionnaires. The clinicians that responded were 372. The calculated response rate was 54.5%.

Two complementary methods of administering the questionnaire were employed: an online survey was used and hard-copy questionnaires were distributed at the time of academic meetings. For the online survey, a link was created and distributed to participants via e-mail. In both cases, participant information and consent sheets were distributed along with the survey questionnaires.

### Data analysis

For the online survey, data were collected on Microsoft Forms. Data from the data collection sheets were entered into a Microsoft Excel spreadsheet. These data were imported into STATA^®^ Version 15 (Stata Corp) for further analysis. Descriptive statistics were conducted.

Data were grouped into categorical and continuous groups. Categorical variables were presented as frequency and percentages. The continuous variables such as the years of using PACS were presented as means ± standard deviation (s.d.) or medians and interquartile range (if not normally distributed). As part of quality assurance, data cleaning processes included checking for duplicates, missing values, recoding and categorising variables. Correlations between categorical data were assessed using the Pearson’s chi-square or Fisher exact test. Pearson’s correlation was done and Cronbach’s alpha was used to check the reliability and validity of the questions.

The open-ended questions were analysed using a method of content analysis that determines the number of times certain qualities appear in a written text. In the context of this study, two coding units were used: words and themes.

### Statistical analysis

Graphs of the results were generated. All statistical analyses were two-sided and *p*-values < 0.05 were statistically significant. For the purpose of using the 2 × 2 chi-square, the four-point Likert scale was collapsed into two categories: disagree included strongly disagree and moderately disagree and agree included strongly agree and moderately agree.

- The degree of level of agreement was further categorised as follows:
  ▪ Strong agreement: 75% – 100%
  ▪ Moderate agreement: 50% – 74%
  ▪ Minimal agreement: 25% – 49%
  ▪ Little agreement: 0% – 24%

## Results

### Demographics

**FIGURE 1:**
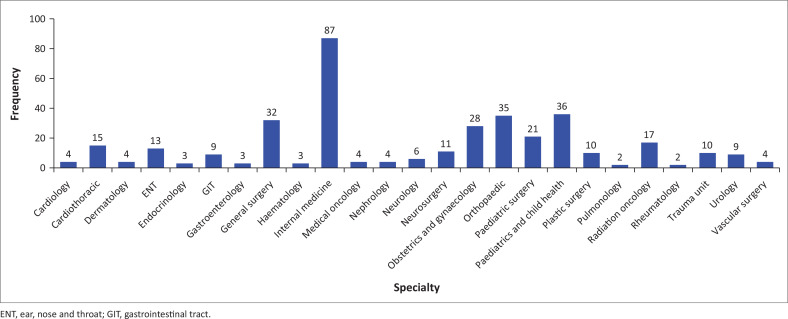
Distribution of clinicians by specialty (*n* = 372).

**FIGURE 2:**
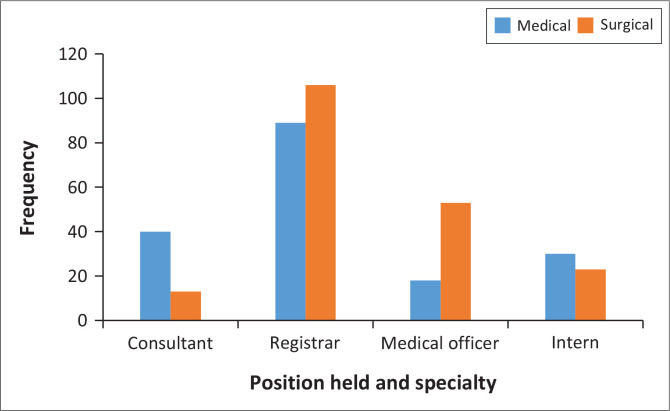
Participants’ distribution according to position held and specialty.

The highest number of respondents were registrars, 194 out of 372 (52%). Distribution of participants by speciality and position held are presented in Figure 1 and Figure 2.

**FIGURE 3:**
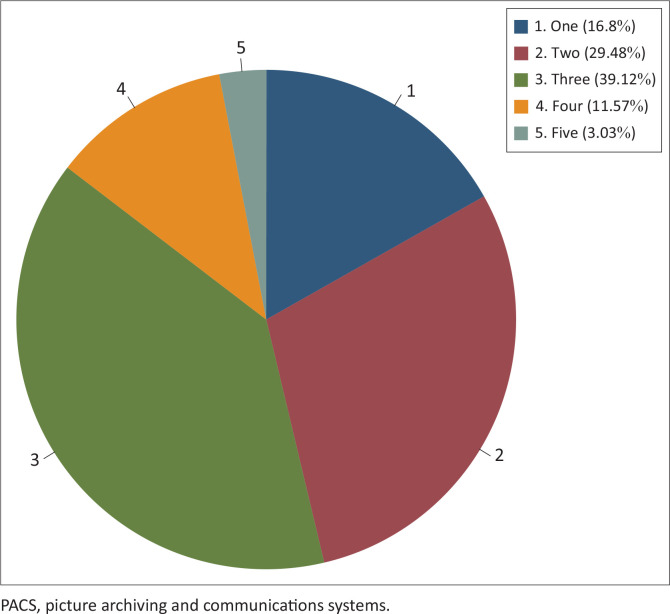
Chart of participants’ years of picture archiving and communications systems experience.

Overall, the mean (s.d.) for using PACS by position held was 2.5 (1.03) years. Consultant mean was 3.38 (0.93) years and interns had the lowest mean of 1.42 (0.75) years. When categorised to the nearest number, the majority had used PACS for 3 years composing 39% of the respondents, and only 3% had used PACS for 5 years. The distribution of participants by years of PACS experience is presented in Figure 3.

The results showed that the majority of respondents accessed PACS for both reports and examinations. Twenty-eight of the 54 (51.9%) interns accessed reports only, while 26 out of 54 (48.2%) accessed both reports and examinations. The majority of consultants 52 out of 53 (98%), medical officers 67 out of 69 (97%) and registrars 183 out of 194 (94%) accessed both reports and examinations. This was statistically significant with *p*-value = 0.001. The vast majority of the respondents accessed PACS from a hospital PC workstation 370 out of 372 (99.5%).

### Survey results

The reliability of the questionnaire was measured using Cronbach’s alpha. The data is presented in Table 1 and Table 2.

**TABLE 1:** Perceived benefits statement reliability tests.

Variable	Mean	s.d.	*r*-Coefficient	Alpha
PACS has reduced the time I must wait to review an exam (images)	3.57	0.52	0.5798	0.7911
I access exams more frequently with PACS than I do with film	3.53	0.54	0.7133	0.7696
PACS has facilitated consultation between myself, other clinicians and/or radiologists	3.40	0.52	0.7242	0.7677
My efficiency has improved because of PACS	3.26	0.99	0.4027	0.8161
PACS has improved my ability to make decisions regarding patient care	3.25	0.47	0.7519	0.7628
PACS has led to a reduction in my patients’ length of stay in hospital	3.38	0.77	0.4957	0.8035
PACS has reduced the number of exams reordered	3.31	0.52	0.6805	0.7750
PACS has enhanced patient care and service delivery at CMJAH	3.52	0.71	0.5360	0.7976
PACS has improved medical student and/or registrar teaching	3.35	0.52	0.7281	0.7670

Note: Alpha greater than 0.7 shows greater reliability for the answers to specific benefit questions. The *r*-coefficients were below 0.9 showing less correlation in questions.

s.d., standard deviation; PACS, picture archiving and communication system; CMJAH, Charlotte Maxeke Johannesburg Academic Hospital.

**TABLE 2:** Perceived challenges statement reliability test.

Variable	Mean	s.d.	*r*-Coefficient	Alpha
PACS produces inadequate image quality on our image review stations	1.84	0.62	0.4264	0.6827
I have difficulty finding images and/or reports when needed	1.85	0.51	0.5375	0.6567
I experience inadequate workstation performance (speed).	2.28	0.67	0.6169	0.6344
I have inadequate access to PACS viewing stations	2.86	0.59	0.4845	0.6682
I have difficulty logging on to the system	2.08	0.59	0.6224	0.6335
PACS downtime is higher than acceptable (malfunction of system)	2.31	0.70	0.6551	0.6233
I have received insufficient training in this new technology	2.23	0.70	0.5913	0.6413
I am unable to view images at the patient’s bedside (PC or mobile devices)	3.32	0.58	0.3841	0.6907
I experience a lack of availability of the system support (From PACS administrators)	2.79	1.03	0.5019	0.6717

Note: Alpha less than 0.7 showed lesser reliability for the answers to specific potential challenges questions. The *r*-coefficients were below 0.9 showing there was less correlation in questions.

s.d., standard deviation; PACS, picture archiving and communication system; PC, personal computer.

#### Perceived benefits

Most clinicians strongly agreed that PACS has reduced the length of patients’ stay at the hospital (83%), improved the ability for decision making regarding patient care (98%), enhanced patient care and service delivery (88%), reduced time taken to review an exam (98%), increased access to more exams than with film (99%), improved teaching of medical students and registrars (98%), improved consultation with other clinicians and radiologists (99%), and reduced the number of repeat examinations (97%). There was moderate agreement that PACS had improved clinicians’ efficiency (65%) (Table 3).

**TABLE 3:** Picture archiving and communication system survey results.

Statement		Strongly agree		Agree		Neutral		Disagree		Strongly disagree	
		*n*	%	*n*	%	*n*	%	*n*	%	*n*	%
1	PACS has reduced the time I must wait to review an exam (images).	208	55.7	159	46.6	3	0.1	2	0.1	0	0.0
2	I access exams more frequently with PACS than I do with film	192	51.6	176	47.3	2	0.1	0	0.0	2	0.1
3	PACS has facilitated consultation between myself, other clinicians and/or radiologists	222	59.7	145	39.0	3	0.1	0	0.0	1	0.1
4	My efficiency has improved because of PACS	86	23.1	157	42.2	50	13.4	69	18.5	10	2.7
5	PACS has improved my ability to make decisions regarding patient care	95	25.5	274	73.7	1	0.1	1	0.1	1	0.1
6	PACS has led to a reduction in my patients’ length of stay in hospital	79	21.2	228	61.3	21	5.6	43	11.6	1	0.1
7	PACS has reduced the number of exams reordered because the exams were not available (lost or located elsewhere) when I needed them	112	30.1	249	67.0	6	1.6	4	1.1	0	0.0
8	PACS has enhanced patient care and service delivery at Charlotte Maxeke Johannesburg Academic Hospital	117	31.5	210	56.5	41	11.0	1	0.1	2	0.1
9	PACS has improved medical students and/or registrars	116	31.2	248	66.7	7	1.9	1	0.1	0	0.0
10	PACS produces inadequate image quality on our image review stations	1	0.1	6	1.6	6	1.6	271	73.0	87	23.4
11	I have difficulty finding images and/or reports when needed	0	0.0	1	0.1	4	1.1	299	80.4	67	18.0
12	I experience inadequate workstation performance (speed).	6	1.6	89	34.0	7	1.9	252	67.7	18	4.8
13	I have inadequate access to PACS viewing stations	19	5.1	275	74.0	5	1.3	63	16.9	8	2.2
14	I have difficulty logging on to the system	4	1.1	27	7.3	7	1.9	308	82.8	26	7.0
15	PACS downtime is higher than acceptable (malfunction of system)	5	1.3	91	24.5	10	2.7	248	66.7	17	4.6
16	I have received insufficient training in this new technology	2	0.1	68	18.2	12	3.2	268	72.0	21	5.7
17	I am unable to view images at the patient’s bedside (PC or mobile devices)	126	33.9	230	61.8	4	1.1	8	2.1	4	1.1
18	I experience a lack of availability of the system support (From PACS administrators).	9	2.4	129	34.7	50	13.4	176	47.3	6	1.6

PACS, picture archiving and communication system; PC, personal computer.

Of the nine benefit measures asked to clinicians with respect to their position held, there were no significant differences in terms of level of agreement with respect to: time taken to review an exam (*p* = 0.272), exams being accessed more frequently with PACS than with film (*p* = 0.114), impact of PACS on improved consultation with other clinicians and/or radiologists (*p* = 0.311), improved ability to make decision making regarding patient care (*p* = 0.343), reduced number of repeat exams (*p* = 0.075), enhanced patient care and service delivery (*p* = 0.075), improved teaching of medical students and registrars (*p* = 0.132), reduced length of patients’ stay at the hospital (*p* = 0.604). There was significant difference among respondents in the percentage agreement with respect to the impact of PACS on improved efficiency (*p* = 0.02) (Table 4).

**TABLE 4:** Perceived benefits survey results.

Variable	Total		Consultant		Intern		Medical officer		Registrar		*p*
	*n*	%	*n*	%	*n*	%	*n*	%	*n*	%	
**PACS has reduced the time I must wait to review (images)**	-	-	-	-	-	-	-	-	-	-	0.272
Agree	367	98	56	96	54	100	70	99	192	99	-
Disagree	2	1	1	2	0	0	1	1	0	0	-
Neutral	3	1	1	2	0	0	0	0	2	1	-
**I access exams more frequently with PACS than film**	-	-	-	-	-	-	-	-	-	-	0.114
Agree	368	98	51	96	54	100	71	100	192	99	-
Disagree	2	1	2	4	0	0	0	0	0	0	-
Neutral	2	1	0	0	0	0	0	0	2	1	-
**PACS has facilitated consultation with other clinicians**	-	-	-	-	-	-	-	-	-	-	0.311
Agree	367	98	51	96	54	100	70	100	192	99	-
Disagree	1	1	1	2	0	0	0	0	0	0	-
Neutral	3	1	1	2	0	0	0	0	2	1	-
**My efficiency has improved because of PACS**	-	-	-	-	-	-	-	-	-	-	< 0.001
Agree	243	65	15	28	51	94	49	69	128	66	-
Disagree	79	21	23	44	1	1	12	17	43	22	-
Neutral	50	14	15	28	2	3	10	14	23	12	-
**PACS improved ability to make patient care decisions**	-	-	-	-	-	-	-	-	-	-	0.343
Agree	369	98	52	98	54	100	70	99	193	99	-
Disagree	2	1	1	2	0	0	1	1	0	0	-
Neutral	1	1	0	0	0	0	0	0	1	1	-
**PACS has led to a reduction in my patients’ LOS in hospital**	-	-	-	-	-	-	-	-	-	-	0.604
Agree	307	82	39	74	54	100	62	87	152	78	-
Disagree	22	6	8	15	0	0	1	2	13	7	-
Neutral	43	12	6	11	0	0	8	11	29	15	-
**PACS has reduced the number of exams reordered**	-	-	-	-	-	-	-	-	-	-	0.075
Agree	36	97	52	98	53	100	71	100	185	95	-
Disagree	6	2	1	2	0	0	0	0	5	1	-
Neutral	4	1	0	0	0	0	0	0	24	12	-
**PACS enhanced patient care and service delivery at CMJAH**	-	-	-	-	-	-	-	-	-	-	0.075
Agree	327	88	42	81	53	980	64	90	168	87	-
Disagree	3	1	1	2	0	0	0	0	2	1	-
Neutral	41	11	9	17	1	2	7	10	24	12	-
**PACS has improved medical students and/or registrars teaching**	-	-	-	-	-	-	-	-	-	-	0.132
Agree	364	97	50	94	54	100	71	100	189	97	-
Disagree	1	1	1	1	0	0	0	0	1	1	-
Neutral	7	2	2	3	0	0	0	0	7	2	-

PACS, picture archiving and communications systems; CMJAH, Charlotte Maxeke Johannesburg Academic Hospital; LOS, length of stay.

#### Perceived challenges

The majority of clinicians strongly agreed that there was inadequate access to PACS workstations (80%) and mentioned inability to view images at the bedside using portable devices (96%). There was minimal agreement that there was inadequate workstation performance speed (25%), higher than acceptable downtime (26%), and the lack of system support availability (37%). There was little agreement that PACS had resulted in inadequate image quality (2%), that they had received inadequate PACS training (19%), that they had difficulty finding images in PACS when needed (1%), or that they had difficulty logging onto the PACS (8%) (Table 3).

Of the nine indicators for measuring perceived challenges, only three indicators were perceived to be significantly different among clinicians with respect to position held. Half of the interns (50%) agreed they received insufficient training, while only 19% of registrars, 7% of consultants, and 4% of medical officers felt this was the case (*p* = 0.001). The lack of availability of system support was identified by 50% of the interns, 44% of registrars, 22% of consultants and 21% of medical officers (*p* = 0.001). Downtime being higher than acceptable was identified by 26% of consultants, 30% of medical officers, 29% of registrars and 9% of interns (*p* = 0.001) (Table 5).

**TABLE 5:** Perceived challenges survey results.

Variable	Total		Consultant		Intern		MO		Registrar		*p*
	*n*	%	*n*	%	*n*	%	*n*	%	*n*	%	
**Inadequate image quality**	-	-	-	-	-	-	-	-	-	-	0.050
Agree	7	2	4	7	0	0	0	0	3	2	-
Disagree	358	96	47	89	53	98	69	98	189	97	-
Neutral	6	2	2	4	1	2	1	2	2	1	-
**Difficulty finding images**	-	-	-	-	-	-	-	-	-	-	0.434
Agree	1	1	0	0	0	0	1	2	0	0	-
Disagree	366	98	52	98	53	98	70	98	191	99	-
Neutral	4	1	1	2	1	2	0	0	2	1	-
**Inadequate workstation performance**	-	-	-	-	-	-	-	-	-	-	0.096
Agree	95	25	11	21	7	13	23	32	54	28	-
Disagree	270	73	40	75	47	87	47	66	136	70	-
Neutral	7	2	2	4	0	0	1	2	4	2	-
**Inadequate access to workstation**	-	-	-	-	-	-	-	-	-	-	0.088
Agree	294	80	36	68	41	77	61	86	156	81	-
Disagree	71	19	17	32	12	23	10	14	32	17	-
Neutral	5	1	0	0	0	0	0	0	5	2	-
**Difficulty logging in**	-	-	-	-	-	-	-	-	-	-	0.289
Agree	31	8	4	8	7	13	2	3	18	9	-
Disagree	334	90	47	88	47	87	68	96	172	89	-
Neutral	7	2	2	4	0	0	1	1	4	2	-
**Downtime higher than acceptable**	-	-	-	-	-	-	-	-	-	-	0.007
Agree	96	26	14	26	5	9	21	30	56	29	-
Disagree	265	71	36	68	49	91	47	70	131	67	-
Neutral	10	3	3	6	0	0	0	0	7	4	-
**Insufficient PACS training**	-	-	-	-	-	-	-	-	-	-	< 0.001
Agree	70	19	4	7	27	50	3	4	36	19	-
Disagree	289	78	47	87	26	48	67	94	150	78	-
Neutral	12	3	3	6	1	2	1	2	7	3	-
**Cannot view images at bedside**	-	-	-	-	-	-	-	-	-	-	0.840
Agree	356	96	51	96	52	98	70	99	183	94	-
Disagree	11	3	2	4	1	2	1	1	7	4	-
Neutral	4	1	0	0	0	0	0	0	4	2	-
**Lack of availability of system support**	-	-	-	-	-	-	-	-	-	-	0.001
Agree	138	37	12	22	27	50	15	21	84	44	-
Disagree	182	49	30	57	22	41	46	65	84	44	-
Neutral	50	14	11	21	5	9	10	14	24	12	-

PACS, picture archiving and communications systems; MO, medical officer.

#### Open ended questions

A total of 66 out of 371 (17.8%) clinicians responded to the open-ended question on additional comments on benefits and challenges. The total number of views expressed were 93. Positive comments were 46, negative comments were 41, and non-relevant were 6. Of the total number of views expressed (*N* = 93), 48% were focused on benefits, whereas 46% mentioned challenges.

Table 6 presents a summary of the comments expressed by respondents. Taking into consideration that some respondents passed more than one comment in their response, the researcher determined if the views expressed were either negative or positive, and documented them as either a benefit or a challenge.

**TABLE 6:** Summary of open-ended question comments (*N* = 93).

Perceived benefits†	*n*	%	Clinicians’ comments		
			Perceived challenges‡	*n*	%
Overall positive comments included terms like: ‘Saves time’, ‘hugely beneficial’, ‘great’, ‘splendid’, ‘perfect’, ‘good’, ‘better’, ‘no more lost films’, ‘timely access’, ‘efficient’, ‘amazing’, ‘way to go’, ‘easy image comparison’, ‘all images in one file’, ‘makes life easy’, ‘easily accessible’, and ‘works well’	46	49.4	Few PACS access workstations	16	17.2
			Lack of advanced image viewing manipulating tools	9	9.7
			Power outage related downtime	8	8.6
			Lack of beside or offsite access to PACS.	5	5.4
			Other	3	3.1
			Neutral comments	6	6.6

PACS, picture archiving and communication systems.

† , Total comments = 46 (49.4%);

‡ , Total comments = 47 (51.6%).

Access to PACS, whether in the clinic environment or in wards, was noted as a major challenge among 17.2% of respondents. This was followed by the lack of advanced image viewing software 9.7%, power outage related downtime 8.6%, and the lack of bedside access to PACS 5.4%.

An open-ended question yielded respondents’ recommendations for improvements on the current system. A total number of 230 out of 372 (61%) responses were received from respondents (Table 7). After subjective categorisation, the total number of views identified as recommended improvements was 466. The most frequent recommendations were: increase PACS access in wards, clinics and bedside with 157 out of 466 (37%), enable PACS access via portable devices with 143 out of 466 (31%), install advanced image viewing software 36 out of 466 (8%), introduce an online booking system integrated with PACS (7%), integrate systems with other hospital PACS or set up a provincial PACS system (5%), increase the number of PACS training workshops and technical support (5%), and offsite access to PACS (4%).

**TABLE 7:** Proposed recommendations for picture archiving and communication systems improvement (*N* = 466).

Proposed improvements	*n*	%
Increase PACS access workstations in wards/clinics/bedside	157	34
Enable PACS access via portable devices	143	31
Install advanced image viewing software MPR, MIP, 3D, virtual bronchoscopy	36	8
Introduce online booking system	34	7
Integrate system with other hospital PACS (Provincial PACS)	24	5.2
More PACS training workshops and technical support	22	5
Offsite access to PACS	20	4
Improve network speed	10	2
Integrate PACS with EMR	5	1
Decrease downtime	5	1
Improve image quality	1	0.2
Connect PACS to a UPS	3	0.6
Archive ECHO, ECG, cardiac catheterisation, and arthroscopy on to PACS	2	0.4
Improve security, offer all users unique login	1	0.2
Other comments: Unknown, nothing, Is there mobile device access?	3	0.6

MPR, multiplanar reformation; MIP, minimum intensity projection; PACS, picture archiving and communication systems; EMR, electronic medical record; UPS, uninterrupted power supply; ECHO, echocardiogram; ECG, electrocardiogram.

## Discussion

### Patient care and service delivery

The strong agreement response on improved patient care and service delivery compares with the high level of agreement observed in other studies. Lenhart^7^ conducted a study on ‘PACS: Acceptance by orthopaedic surgeons’ wherein she recorded 64% agreement that PACS improved patient care. There are no specific studies in the literature that specifically focus on the impact of PACS on improving patient care. It is difficult to come up with an objective measure for patient care. Watkins^8^ concluded that there was no clearly discernible influence of PACS on clinical decision making; however, prompt access to images could have some beneficial impact. This is particularly the case in ICU and the emergency department where immediate access to images is thought to be more critical in influencing further patient management.

### Reduced hospital length of stay

It can be hypothesised that prompt access to radiology reports and exams via PACS may result in prompt decision making and initiation of treatment, thereby reducing the patient’s length of stay. In a study conducted in Saudi Arabia evaluating PACS at three ministry hospitals by Alalawi et al.,^9^ 79% of the participants agreed with this statement. However, in another study evaluating the benefits of PACS, Bryan et al.^10^ concluded there was no convincing evidence that PACS reduced the length of inpatient stay. This was further supported by a study conducted by MacDonald et al.^3^ who concluded that the length of stay was not significantly impacted by PACS. They pointed out many external influencing factors to PACS such as clinician practice, hospital type and policy, and patient comorbidities.

Although our local clinicians communicated a reduced hospital stay with PACS, examples of some external factors include the following: Charlotte Maxeke Johannesburg Academic Hospital is overburdened by many emergency cases resulting in a lack of availability of high care or intensive care unit (ICU) beds which further delays scheduling of some major elective cases that require post-operative admission to these units. Some of the equipment required for surgical procedures is outsourced from private companies, for example, the equipment for neuro-monitoring and neuro-navigation; however, if these companies are completely booked, there may be a delay in the scheduling of neurosurgical procedures, increasing the hospital length of stay.

### Consultation with other clinicians and impact on efficiency

The authors expected PACS to reduce the interaction between clinicians and radiologists due to the availability of images and reports at multiple sites within the hospital. This study’s results strongly supported this argument: 98% of clinicians agreed that PACS had facilitated consultation among clinicians, and clinicians with radiologists. A limitation of the study is that consultations among clinicians themselves, and consultations between clinicians and radiologists were not separated. This question should have been split into two to specify the type of consultation. MacDonald et al.^3^ documented reduced in-site consultations with radiologists, and increased offsite consultations between radiology and clinicians in a provincial PACS-based system study. There was moderate agreement of 66.4% that PACS had increased offsite consultations. Redfern et al.^11^ supported the notion that the availability of PACS stations at clinical areas would lead to decreased consultations with radiology. Most clinicians suggested they saved time by no longer consulting with the radiology department to view images and/or reports. The radiology exams were readily available at multiple clinician workstations immediately after the images were acquired. Treatment planning could commence prior to the patient’s return from the radiology department. This benefit was particularly observed in the emergency medicine and trauma units.

### Impact on academic teaching

Regarding the impact of PACS on the teaching of medical students and registrars, there was moderate agreement of 67% that there was an improvement. These results correlate with the study by Jorwerkar et al.^1^ where 51% of the respondents were in agreement. Picture archiving and communication systems are valuable for teaching due to the ease with which images can be compared, the convenience with which exams can be archived for use in teaching, and the ease with which image quality may be manipulated.

### Perceived challenges

The challenges most often cited were the inability to view the images at the bedside, the lack of portable device access, and few available viewing stations. While this limitation could be a gap in the implementation plan, it must be analysed within the context of what is practical in the hospital setting of interest. It would be costly to set up workstations at every bedside and in a public sector hospital in a low- to middle-income country, logistically near impossible. Theft of equipment was highlighted as a challenge by the PACS administrators. One practical solution would be for clinicians to access PACS from their portable devices (tablets, laptops and mobile phones). This would reduce the capital cost of deploying more workstations.

### Image quality and performance (speed)

Although image quality assessment is subjective and dependent on the viewing platform, the majority of the respondents were satisfied with the image quality. Only 2% of respondents stated that PACS produces inadequate image quality. Although entry level clinician workstation monitors are not held to the strict quality control standards of dedicated diagnostic display units used for formal radiology reporting, recent technological advances yield these monitors sufficient for general hospital-wide image review. Mobile device technology has certainly matured significantly for use by radiologists in the on-call, hospital offsite setting and by doctors at the bedside or in the operating theatre.^12^ Slow image retrieval can be attributed to network speed which is more of an information technology (IT) support issue, although no formal assessment of this factor was done. Furthermore, the recent electricity supply challenges faced by the country affected the network connectivity and speed.

### System support and training

Insufficient PACS training was reported by 24% of responding clinicians, and 36% agreed that they experienced a lack of system support from PACS administrators. Although 20% – 40% of the respondents did not constitute a majority, this nonetheless suggests there are training and support issues to be addressed. Picture archiving and communication systems administrators conduct two training workshops every year, however, the turnout of clinicians during these PACS training workshops is usually low. This could in part explain why some clinicians felt that they did not receive adequate training. A limitation of the study is the fact that the roles of IT support and PACS administrators were not distinguished when it came to system support.

### Improvements

The most frequent recommendations 320 out of 466 (68%) were related to PACS access. Very few clinicians were aware that PACS can be accessed via portable devices (personal tablets, laptops and cell phones) from within the hospital. The hospital PACS is wired through a local area network (LAN) and is web-based. The hospital already has the infrastructure to facilitate wireless connectivity to this network. Devices to be connected to this network will need to be configured by the IT department. Some doctors opined that they were able to access laboratory results from their portable devices through internet connection; hence, the same technology could be availed for PACS access. One respondent commented that the network is very slow on portable devices.

Offsite access will be beneficial to clinicians who are on call as they will be able to view images in the comfort of their homes or call rooms. Furthermore, this will benefit those who would want to access images for teaching purposes on virtual platforms. This will require an upgrade to a private cloud-based PACS which is more cost-effective, reliable and secure.^13^ Off-site access to PACS is a challenge for onsite PACS systems due to security and privacy requirements; therefore, onsite PACS solutions have trouble transmitting secure data outside of their immediate area.^14^ Contrarily, cloud PACS is designed for offsite access and providers follow stringent security and privacy guidelines.^14^ Budget constraints, funding, current PACS vendor’s contractual obligations and government decisions may influence the hospitals’ decisions on which PACS system to upgrade to.

Few comments recommended the deployment of a provincial PACS (24 out of 466, 5%). This will significantly reduce the number of repeat exams, as well as the number of unnecessary patient transfers as a result of prior image review and consultations. The referring hospitals connected to CMJAH PACS include Berta Gxowa, Far East Rand, Sizwe and Pholosong hospitals. At the time of writing, efforts were being made to connect Leratong, Helen Joseph and Yusuf Dadoo hospitals. The downside of this connectivity is that it is one way, with only CMJAH being able to access studies done from the connected hospitals. Clinicians from referring hospitals are unable to access the CMJAH PACS. The iSite archive could not be integrated into the other hospital archives as they use different running software. These have different repository archives, architectures and data registry. Charlotte Maxeke Johannesburg Academic Hospital has computers installed with software compatible with the referring hospitals, allowing it to access studies done at these hospitals.

Most clinician PACS workstations are equipped with basic image manipulating tools, which include zoom, panning, measure, window and level function. Some clinicians highlighted the need to have more advanced hanging protocols and image manipulation tools similar to those found in the radiology PACS workstations. These include 3-D reconstruction, multiplanar reformation (MPR), virtual bronchoscopy, virtual colonoscopy and angiography post processing techniques for planning endovascular aneurysm repair. It is impractical to equip every clinician PACS station with advanced image viewing software due to the cost involved. The solution would be to customise the image viewing software according to speciality requirements.

Only 7% of comments referred to an online booking system for radiology exams. This recommendation came mainly from interns, medical officers and registrars. Most of these respondents suggested this will increase their efficiency as they will spend less time going to the radiology department. From a radiology department perspective, physical bookings have the advantage of planning a patient’s imaging in consultation with the requesting clinician, which will aid coming up with the best modality suitable for the clinical question and ultimately reduce unnecessary bookings. Telephonic discussions and online booking systems are compelling options to consider in the post-coronavirus disease 2019 (COVID-19) milieu, where everything has moved to digital platforms and remote access.

In this study, issues raised regarding downtime were specifically related to power cuts rather than routine scheduled maintenance. At the time of writing, South Africa was experiencing severe power outages; this crisis is predicted to continue into the near future. Connecting the entire PACS infrastructure to an uninterrupted power supply (UPS) system was suggested as an option to mitigate downtime. The radiology department prints hard copy films for clinicians during PACS downtime. Uninterrupted power supply (UPS) connectivity will help minimise film printing costs.

## Limitations

- The study was limited to a post-PACS implementation evaluation. To fully assess the impact of PACS on clinical practice, a study that involved the pre- and post-implementation would have been ideal. However, this was not feasible as there were few respondents from the pre-implementation era.
- Despite a reasonable sample size, the response rate was low at 54%. At the time of writing, the hospital was only partially open due to ongoing renovations after a fire incident which forced the entire hospital to close in 2021. Some departments were still not fully functional with their staff deployed to satellite hospitals. This could have contributed to the low response rate.
- Only clinicians referring patients to the radiology department were included. The study excluded radiologists. Hence, there is a need for further research to validate research findings by comparing outcomes of PACS users working in different environments.
- During data analysis, detailed information could have been lost by collapsing the four-point Likert scale to two categories, which were ‘disagree’ and ‘agree’.

## Conclusion

The findings of this study provide overwhelming evidence that referring clinicians support the implementation of a hospital-wide PACS. The benefits of PACS, in particular reduction of repeat imaging, ease of comparison with previous imaging, image and report availability at multiple sites at any time and eliminating the scenario of lost films were seen as compelling rationale for the implementation of a hospital-wide PACS system.

The main challenges raised regarded PACS access both at inpatient and outpatient environments, downtime and the lack of advanced image manipulating tools at clinician workstations. These issues were cited as major areas that need improvements for clinicians to fully realise the benefits of PACS. The case for switching to a cloud-based PACS system is strong given the acknowledged desire from clinicians for offsite access and the difficulties faced by CMJAH with regard to equipment theft, few PACS access stations and frequent power outages.

This study will serve as a benchmark for future hospital and provincial-wide PACS deployment projects in public hospitals.
